# Manual tracking for solar parabolic concentrator – For the case of solar injera baking, Ethiopia

**DOI:** 10.1016/j.heliyon.2023.e12884

**Published:** 2023-01-10

**Authors:** Arkbom Hailu Asfaw

**Affiliations:** a Sustainable Energy Center of Excellence, Addis Ababa Science and Technology University, P.O. Box 16417, Addis Ababa, Ethiopia; b Nuclear Technology Center of Excellence, Addis Ababa Science and Technology University, P.O. Box 16417, Addis Ababa, Ethiopia; c Department of Environmental Engineering, College of Biological and Chemical Engineering, Addis Ababa Science, Ethiopia

**Keywords:** Manual solar tracking system, Altitude and azimuth angle, Parabolic collector, Solar parabolic injera stove

## Abstract

The efficiency of renewable energy equipment is highly reliant on the mechanism for properly capturing the resource and the equipment's performance. For any solar-powered device tracking mechanism is critical; it must be able to follow the pattern of the sun's path. This research created a manual tracking mechanism for a solar-powered steam injera stove. The design of the tracking mechanism tracks the path of the sun seasonally and daily for 5 h, half in the morning and a half in the afternoon, beginning at solar noon, it tracks the sun's path every 10 min. It investigates the relationship between solar intensity and receiver surface temperature. There were two experimental tests, one with one receiver surface data point and the other with three data points. The correlation coefficient of solar intensity with heat temperature on the receiver surface was r = 0.726 at the first data point. And, for the three points where data was collected for one and a half hours in the afternoon, on three-point of the receiver surface lower points, the correlation of solar intensity and average heat temperate of those three points is r = 0.766. For both of the experiment, the relationship of the solar intensity pattern shows there is a strong positive correlation with the temperature on the surface of the receiver which indicates the manual tracking system move with the proper path of the sun and concentrates the sun on the point receiver of the solar injera stove.

## Highlights

• Proper tracking mechanism significantly enhance the performance of a solar parabolic dish.
• Daily tracking and seasonal tracking will significantly alter efficient of point receiver.
• For tropical region horizontal daily tracking is more important than vertical seasonal tracking.

## Graphicalabstract

**Figure:**
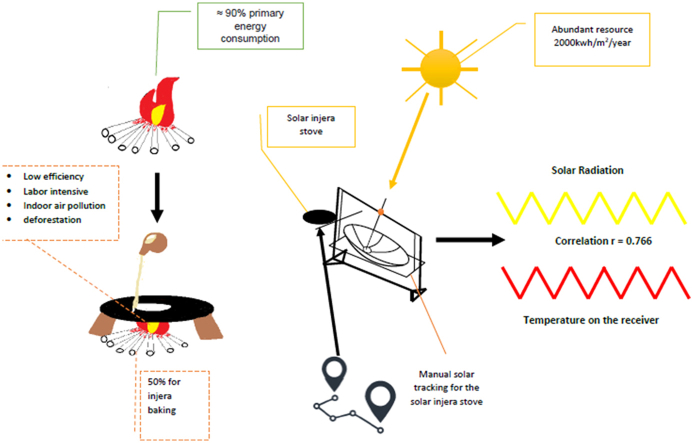

## 1 Introduction

Utilizing solar radiation to meet the energy needs of a location where there is an abundance of solar radiation, particularly in a tropical region, is an unavoidable option. In Sub-Saharan Africa, traditional biomass dominates primary energy consumption, putting pressure on the region not to maintain its natural balance, resulting in a climate problem [1,2]. Cooking consumed the majority of the energy in this area, causing an additional environmental problem due to indoor air pollution [2]. Because solar energy is abundant in this area, it is the best solution for replacing this energy demand.

As part of Sub-Saharan Africa, Ethiopia exhibits the same energy profile and problem, with biomass accounting for 90% of total energy consumption, with half of this energy going toward baking the sole flat thin plate bread known locally as injera [2,3]. Most Ethiopians consume injera on a daily basis, which is baked using various energy sources, the majority of which are biomass with low energy conversion percentages ranging from 6 to 12 energy efficient and 20 to 33% biomass fuel saving stove as compare to open three stone stove [[4], [5], [6]]. Cooking with renewable energy has been attempted using solar energy and some modified technology using biomass as an energy source [7,8] Solar injera baking systems can significantly help to improve the country's natural deterioration by replacing traditional baking energy consumption. At the same time, it reduces the labor force and the negative impact of indoor pollution on women. This study examines the manual tracking mechanism of this system, which will significantly improve its performance.

## 2 Tracking systems

The energy production of any solar-based equipment is highly reliant on the tracking mechanism, which is exacerbated when it is a point concentrator dish, where the reflected solar ray is concentrated on a point receiver. Daily tracking and seasonal tracking are the two types of tracking. Daily tracking moves the collector's position from west to east so that it will track the movement of the sun from east to west along the north south axis, whereas seasonal tracking follows the sun's movement from north to south and vice versa along the east west axis of rotation. The mechanism by which the collector tracks the sun can be either manual or automatic. Automatic tracking necessitates a power source and gear that assists in moving the collector along the path of the sun; additionally, the alignment between the position of the sun and the receiver will be adjusted using a light sensor and it enhance the performance of the power producing part 30–40% more than that of stationary collector [[9], [10], [11], [12]] Manual tracking, on the other hand, does not require any power source to track the sun path; instead, it will be aided by man power using some designation for each time of day to align the position of the collector surface area with a normal angle to the position of the sun [13,14]. The receiver for a parabolic trough and concentrated solar power must be in a concentric point between the sun and the center of the parabolic trough line or concentrator point receiver respectively.

**Fig. 1:**
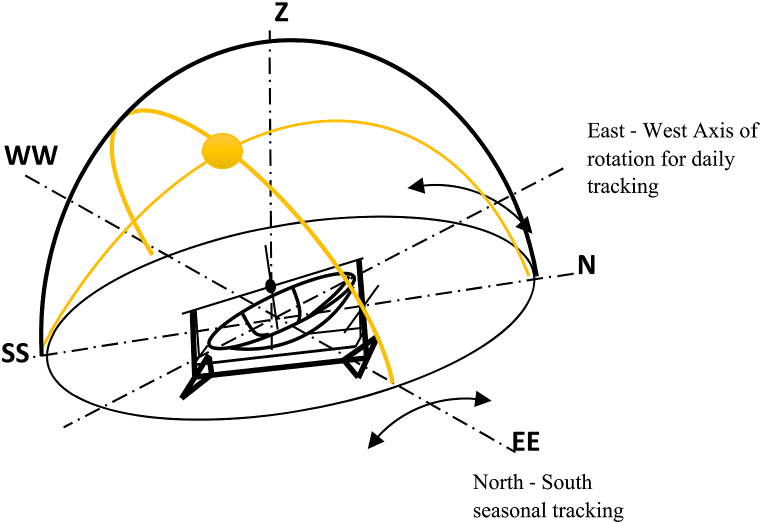
Double Axis tracking of the system.

Single axis tracking can be done daily or monthly, but when they are combined, it is called double axis tracking [15,16] as shown explicitly in Fig. 1. For tropical areas, horizontal daily tracking is more important than vertical seasonal tracking. On the contrary, vertical seasonal tracking is far more vital in polar regions [15,17].

### 2.1 Manual tracking mechanism

**Fig. 2:**
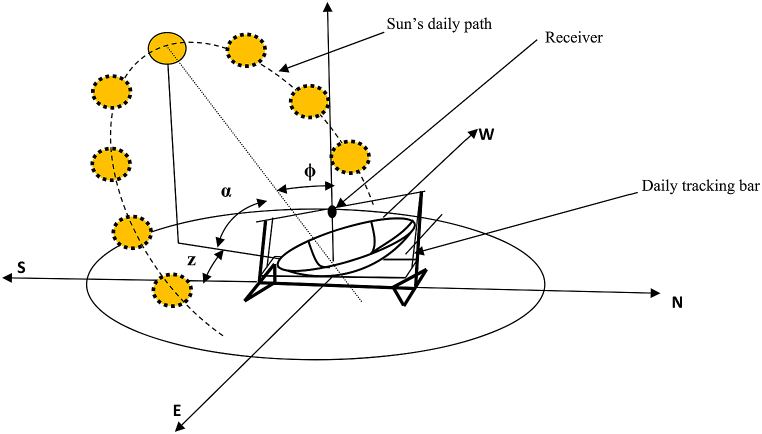
Apparent daily path of the sun across the sky from sunrise to sunset on the parabolic dish.

**Fig. 3:**
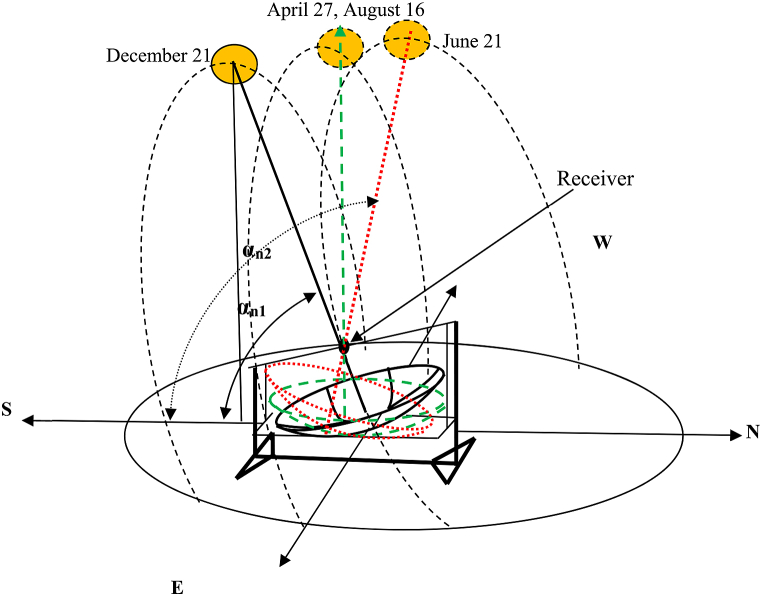
Annual changes in the sun's position in the sky on the laboratory set up.

The parabolic dish used in this experiment was set up to track the sun beam along two axes. The first tracking system is for daily solar radiation from east to west (see Fig. 2), while the second is for monthly solar radiation angle fluctuation (see Fig. 3), in which the concentrator's location shifts from north to south and vice versa. The experiment area had a latitude of 13.5°, and the days of the year when the sun will be overhead will be April 27 after the winter time and August 16 during the summer time, as calculated using Eqs. (3) and (4).

**Figure:**
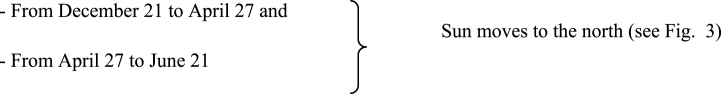

So, the center of the concentrator dish will make an angle that is more than 900, the solar noon altitude angle that is created by the ray of the sun and the horizontal plane in the north-south direction will be more than 900 (αn2) (see Fig. 3) while the sun is moving to the end point in the northern hemisphere at June 21 and.

**Figure:**
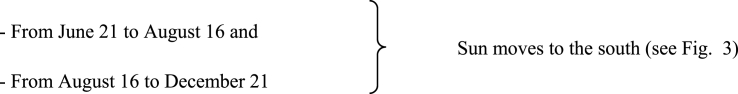

On the second round, particularly when the sun moves from July 21 to August 16 and to December 21 the solar noon altitude angle will become lower than 90° with the horizontal plane (α_n1_).

The sun's most visible motion is that it travels in an arc across the sky every day, reaching its highest point around mid-day (see Fig. 2). The sunrise and sunset points shift northward over the horizon as winter gives way to spring and ultimately summer. The days become longer in the Northern Hemisphere as the sun rises earlier and sets later each day, and its route rises higher in the sky. The sun is at its most northerly point with regard to the earth on June 21, which is known as the summer solstice, and daytime is at its peak for northern hemisphere nations on this day. Even though the northern hemisphere's maximum day time is in the end of June, Ethiopia's rainy season begins after this month for two months, and the sunlight hour is at its lowest during this month and thereafter, for Mekelle as well where the experiment is conducted. On December 21, the winter solstice, six months later, the opposite is true, as the sun is at its farthest southerly point (see Fig. 3),

The following formulas are used to calculate the angle for daily and monthly tracking variation.

#### 2.1.1 Daily solar tracking

**Fig. 4:**
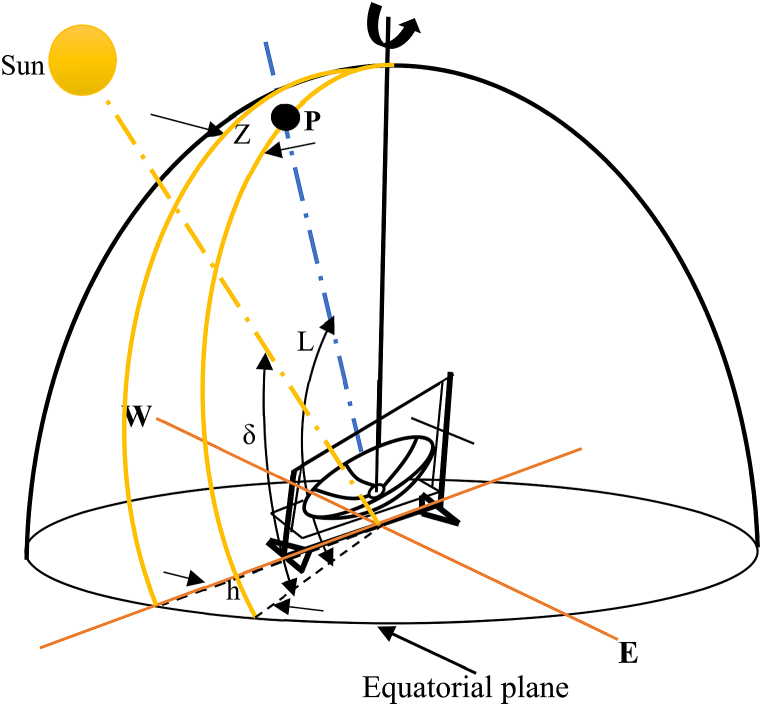
Definition of latitude, hour angle and solar declination.

For daily tracking, the solar hour angle, defined as the angle through which the earth would revolve to bring the meridian of the point directly under the sun, is required (see Fig. 4). The hour angle or azimuth angle is zero at local solar noon, with each 360/24 or 15° of longitude equating to 1 h [18]. The hour angle in degrees is metaphorically expressed with Eq (1):

(1) h=±0.25(Numberofminutesfromlocalsolarnoon)

where, the pulse sign applied to afternoon hours and the minus sign to morning hours.

#### 2.1.2 Monthly solar tracking

Monthly solar tracking requires calculating the solar altitude angle so that the relative location of the concentrator's center with the sun ray and the earth's horizontal plane will be determined for each month. The solar altitude angle has a mathematical expression as depicted in Eq. (2), [19]:

(2) Sin(α)=Cos(φ)=Sin(L)Sin(δ)+Cos(L)Cos(δ)Cos(h)

where L = latitude of the place, in this case for Mekelle, L-13.5°, h = hour angle, described on Eq. (1), δ = Declination angle, it is the angle between the sun-earth centerline and the projection of this line on the equatorial plane, expressed in Eq. (3).

The following equation can be used to compute the earth's declination angle on any given day of the year [19].

(3) $$\delta =23.45\;\mathrm{Sin}\left[\frac{360}{365}\left(284+N\right)\right]$$

where N – Number of the day of that particular tracking day in a year, starting from 1st of January.

The sun is exactly on the meridian, which contains the north-south line, at solar noon, and thus the solar azimuth is 0° as it can clearly see from Fig. 4, [19]. As a result, the noon altitude $\alpha_{n}$ is described in Eq. (4)

(4) $$\alpha_{n}=90{}^{\circ}-L+\delta$$

The above formula can be identified, using Fig. 4 this is a complement of optimal tilted angle (γ) which is the difference of latitude and declination angle [20]. According to the above formula, for Mekelle city the maximum and minimum noon altitude angles for latitude of 13.50 N and sun declination of 23.450 and −23.450 respectively are αn1 = 53.050 and αn2 = 99.950. This means the central axis of the solar parabolic dish concentrator will make a maximum of 36.950 to the south and 9.950 to the north from the vertical axis (see Fig. 12). For each fifteen days of the year the altitude angle is calculated as it can be clearly seen in Table 2.

## 3 Solar injera stove orientation, testing equipments and procedure

**Fig. 5:**
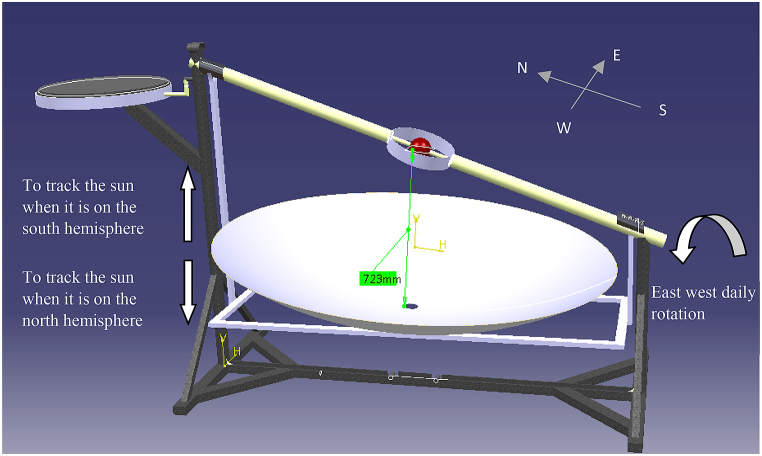
Solar injera stove with manual tracking.

The body designed for solar energy production to bake injera is shown below (Fig. 5). The collector aperture area is 1.8 m in size, with a depth of 0.28 m and a focal length of 0.723 m. The concentrator has a fixed focal length to focus incident solar radiation on a fixed receiver. For daily rotation, the concentrator's axis of rotation is along the true north south side, and for monthly tracking, the concentrator can move to the north or south along the east west axis, as shown in Fig. 5.

### 3.1 Testing equipment

The setup is tested by comparing the temperature on the receiver and the solar radiation on the concentrator to determine the exact correlation of the temperature obtained on the receiver and the intensity of the solar radiation.

The data gathered during the experiment are listed below.

o Temperature readings at various points on the receiver.
o Solar radiation intensity on the concentrator's surface.

Different spots on the receiver's surface were chosen to determine the temperature differential at different points of the receiver so that the position of the point relative to the sun as well as the reflector could be evaluated. On the first experiment, one point was taken to compare with that day's solar radiation, and three points were taken on the second experiment to see the relative effect of the sun position as the reflector rotated, and then on those three points on the receiver. Temperature measurements were taken using a K-type thermocouple with data logger and the Lab VIEW Signal Express 2009 software on a laptop.

The solar radiation of the testing day was recorded at 5- and 10-min intervals, and the temperature variation on the receiver's surface was recorded at 1 s intervals, but this data can be compressed to 1 min, 5 min, and 10 min to see the temperature variation that we were looking for.

The data collecting equipment for evaluating the solar radiation and temperature are as follow.

**Fig. 6:**
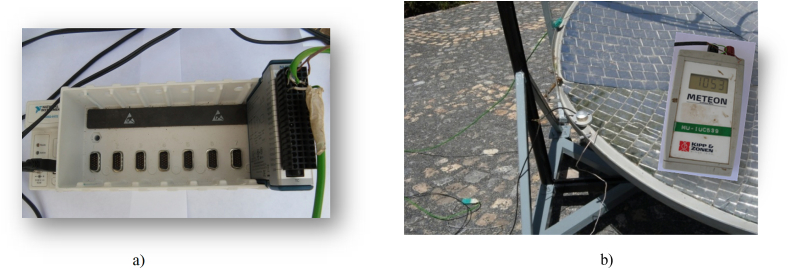
Testing equipments (a) National Instrument Data logger (b) pyranometer with its display.

**Fig. 7:**
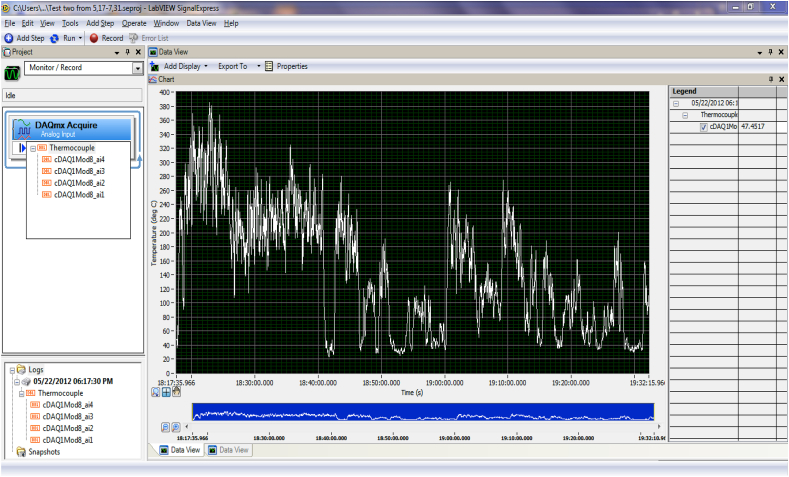
Lab VIEWS signal express 2009 environment.

• METEON Pyranometer with its display to record the daily horizontal solar radiation on the concentrator parabolic dish, (METEON Irradiance meter) (see Fig. 6b).
• Data logger to send the data from k-type thermocouples to the laptop using Lab VIEW Signal Express software, Lab VIEW Signal Express 2009 software to gather temperature data measurements on different points of the receiver (see Figs. 6a and 7).
• K-type thermocouple with extension cables.
• Laptop.

The majority of the equipment used in this experiment was digital for data accuracy, and a data logger with software that can interface with any laptop was used for temperature reading on the system for easy data processing and manipulation.

Every temperature reading from the solar injera baking receiver was recorded on a data logger, which was connected to a laptop via the National Instruments Lab VIEW Signal Express 2009 software for Data logger Laboratory. The Lab VIEW Signal Express software displays the temperature profile of the selected spot using the thermocouple input via its user-friendly interface, as shown in Fig. 7.

The data can be collected over a variety of time intervals; with this software, the data is collected in less than 1 s intervals, but the time interval represented in the above Fig. 7 is for 10 s intervals, allowing the temperature fluctuation to be seen over this time span.

## 4 Results and discussion

### 4.1 Building laboratory setup with manual tracking

A parabolic solar concentrator is the best solution for collecting a large amount of solar radiation from the sun. This is because parabolic solar concentrators have two axes of solar tracking and can provide indicative temperatures ranging from 100 °C to 1500 °C depending on the solar intensity, aperture area, and other variables [19].

The procedure that was followed to execute the task was:

1) Design of the manual tracking that follows the exact path of the sun taking into account the location latitude.
2) Examine the correlation between the temperature on the receiver and the variation of solar radiation.

**Fig. 8:**
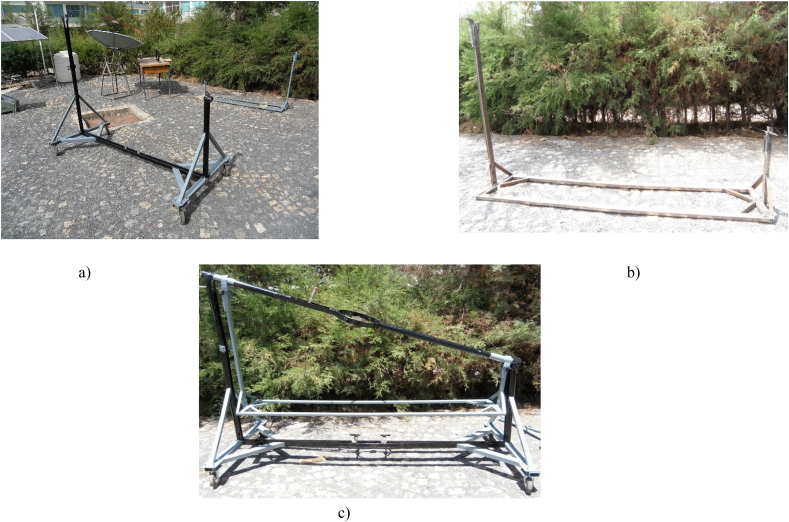
(a) A metal support for manual tracking and concentrator dish (b) Manual tracker structure (c) Assembly of the tracker with the whole structure.

In this study a support of a reflector that aid in tracking the collector for daily and seasonal variations was designed and manufactured (Fig. 8b). The structure will rotate along the axis where the receiver is installed (Fig. 8a and c). Because the experimental area is located in the north hemisphere, the structure that supports the reflector rotates along the true north south axis with the concentrator facing the true south face most of the time of the year. For seasonal variation, the concentrator revolves along the east-west axis, with the line of rotation passing through the receiver's point.

When the whole system assembled, it looks like Fig. 5. The whole system was constructed in order to have a good strength to handle a high pressure in the closed stainless-steel tube and support the concentrator and injera baking clay plate. This structure can be transported from place to place without any difficulty by disassembling the concentrator, the piping system, the external support structure and the solar injera stove. The external structure is equipped with four wheels that allow it to be moved from one location to another see (Fig. 8a and c).

#### 4.1.1 Manual tracking mechanism

The structure that carries the concentrator dish in a fixed position with the dimension of the focal length from the concentrator center to the center of the receiver and support the load of the concentrator dish with the mirror attached to it was designed first for daily and monthly tracking. Using these two concepts, a support structure made of 40 mm × 20 mm rectangular tubular iron with a thickness of 1.5 mm was built, as shown in Fig. 8.

#### 4.1.2 Daily manual tracking

The angle variations for daily tracking were calculated using the minutes variation of a day, which means that according to Eq. (1), the sun will rotate 15° within 1 h (60 min), or 1° rotation of the sun will be created within 4 min. As a result, if we want to track for 4 h a day, we need 60° rotations of the concentrator on the inclined pipe in which the receiver is mounted with Stainless Steel (SS) pipes. Taking the preceding idea into account, the angle of tracking on the system was calculated using the system's geometer, as shown below.

**Fig. 9:**
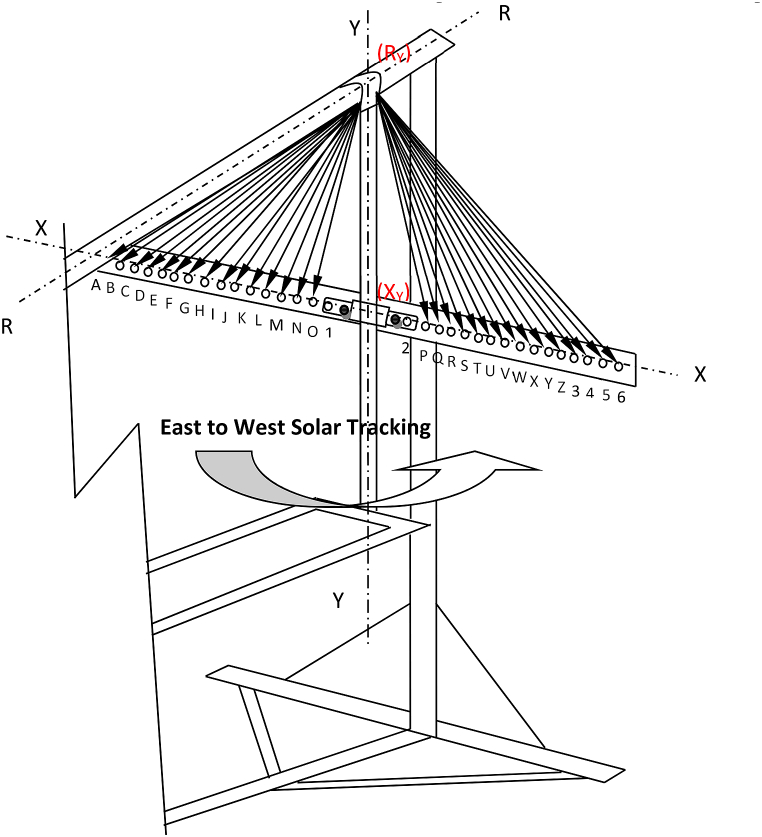
Holes for Morning and Afternoon Manual tracking.

For solar noon, the reflector's position will be zero degrees, with the axis of rotation aligned with the line Ry (intersection of the Radial axis and the Y axis) - Xy (intersection of the X axis and the Y axis) or along the Y axis as it can be clearly seen from Fig. 9. The angle calculations for morning tracking, left side from “A” to “0” have been calculated, and a similar concept has deployed to calculate afternoon tracking from “P” to “6”, as shown in Table 1 and Fig. 9. The vertical hanging structure measures 600 mm from the radial axis (Ry) to the X axis, where the manual tracking hole is located. As a result, this can be considered as a vertical distance to calculate the horizontal hole to each time of day, the horizontal hole for each 10-min variation or 2.5-degree variation can be calculated using the angle variation and the vertical distance, as shown in Table 1.

**Table 1:** Horizontal hole dimension and Angle variation for daily solar tracking.

Point on horizontal bar for Morning tracking	Morning time	Morning hour angles	Y (vertical length), mm	X for 10 Minutes variation (mm)	Angle (degree)	Solar Angle variation for 10 min (degree)	Afternoon hour angles	Afternoon time	Point on horizontal bar for Afternoon tracking
RY-XY	12:00	90	600	95.00	9	0	90	12:00	RY-XY (1,2)
O	11:50	−87.5	600	122.07	11.5	2.5	87.5	12:10	P
N	11:40	−85	600	149.60	14	5	85	12:20	Q
M	11:30	−82.5	600	177.73	16.5	7.5	82.5	12:30	R
L	11:20	−80	600	206.60	19	10	80	12:40	S
K	11:10	−77.5	600	236.35	21.5	12.5	77.5	12:50	T
J	11:00	−75	600	267.14	24	15	75	13:00	U
I	10:50	−72.5	600	299.15	26.5	17.5	72.5	13:10	V
H	10:40	−70	600	332.59	29	20	70	13:20	W
G	10:30	−67.5	600	367.68	31.5	22.5	67.5	13:30	X
F	10:20	−65	600	404.71	34	25	65	13:40	Y
E	10:10	−62.5	600	443.98	36.5	27.5	62.5	13:50	Z
D	10:00	−60	600	485.87	39	30	60	14:00	3
C	9:50	−57.5	600	530.84	41.5	32.5	57.5	14:10	4
B	9:40	−55	600	579.41	44	35	55	14:20	5
A	9:30	−52.5	600	632.27	46.5	37.5	52.5	14:30	6

**Table 2:** Vertical distance of the parabolic dish upper edge from the horizontal hanging bar for monthly tracking of solar radiations.

Week	Number of days	6tion Angle (degree)	Latitude of Mekelle (degree)	Noon altitude angle (degree)	Angle with the vertical Axis (degree)	Vertical distance, Y (mm)	Vertical Distance from zero reference (mm)
1-Jan	1	−23.01	13.5	53.49	36.51	1243.32	962.55
15-Jan	15	−21.27	13.5	55.23	34.77	1167.46	886.68
1-Feb	32	−17.52	13.5	58.98	31.02	1022.16	741.38
15-Feb	46	−13.29	13.5	63.21	26.79	881.76	600.99
1-Mar	60	−8.29	13.5	68.21	21.79	739.31	458.54
15-Mar	74	−2.82	13.5	73.68	16.32	604.36	323.58
1-Apr	91	4.02	13.5	80.52	9.48	457.63	176.86
15-Apr	105	9.41	13.5	85.91	4.09	353.69	72.91
27-Apr	116.5	13.45	13.5	89.95	0.05	280.77	0.00
1-May	121	14.90	13.5	91.40	−1.40	255.47	−25.31
15-May	135	18.79	13.5	95.29	−5.29	189.00	−91.77
1-Jun	152	22.04	13.5	98.54	−8.54	134.95	−145.82
15-Jun	166	23.31	13.5	99.81	−9.81	114.00	−166.78
1-Jul	182	23.12	13.5	99.62	−9.62	117.17	−163.60
15-Jul	196	21.52	13.5	98.02	−8.02	143.57	−137.20
1-Aug	213	17.91	13.5	94.41	−4.41	203.83	−76.94
15-Aug	227	13.78	13.5	90.28	−0.28	274.99	−5.78
16-Aug	228	13.45	13.5	89.95	0.05	280.77	0.00
1-Sep	244	7.72	13.5	84.22	5.78	385.33	104.56
15-Sep	258	2.22	13.5	78.72	11.28	494.38	213.61
1-Oct	274	−4.22	13.5	72.28	17.72	637.06	356.28
15-Oct	288	−9.60	13.5	66.90	23.10	774.50	493.73
1-Nov	305	−15.36	13.5	61.14	28.86	948.00	667.22
15-Nov	319	−19.15	13.5	57.35	32.65	1082.57	801.80
1-Dec	334	−21.97	13.5	54.53	35.47	1197.24	916.46
15-Dec	348	−23.29	13.5	53.21	36.79	1256.10	975.33

*The negative angle is the angle formed by the parabolic vertical axis and the system vertical axis on the north side, while the positive angle is on the south side. And the negative vertical distance from zero reference is the dimension below the horizontal parabolic position.

The first solar noon hole is located 9° and 95 mm from the vertical axis on each side of the Y axis (point 1 and 2). The angle and distance calculation will begin here, and each hole will be pierced for every 10 min in the morning and afternoon using this reference, as stated explicitly on Eq. (5).

(5) (Xy−P)/600=Tan(9+2.5)

**Fig. 10:**
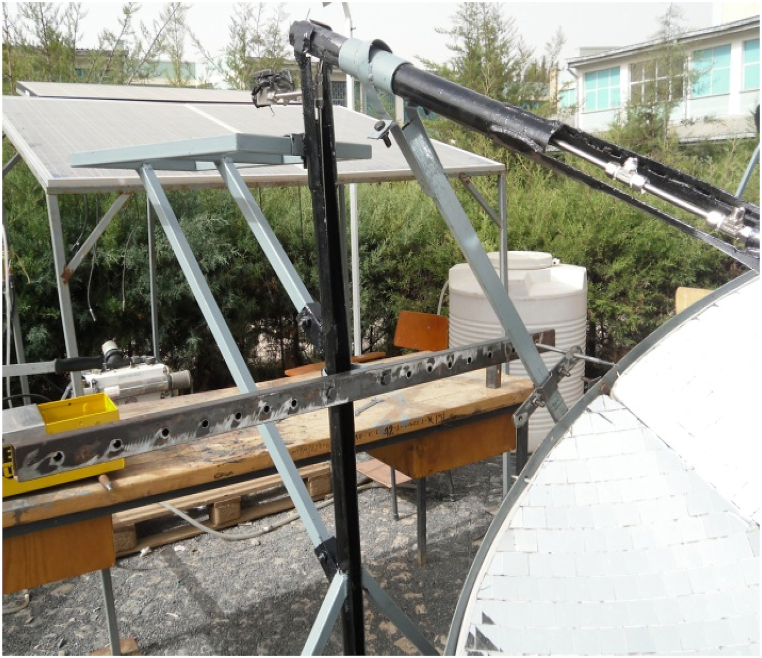
Horizontal bar with 10 min interval holes.

**Fig. 11:**
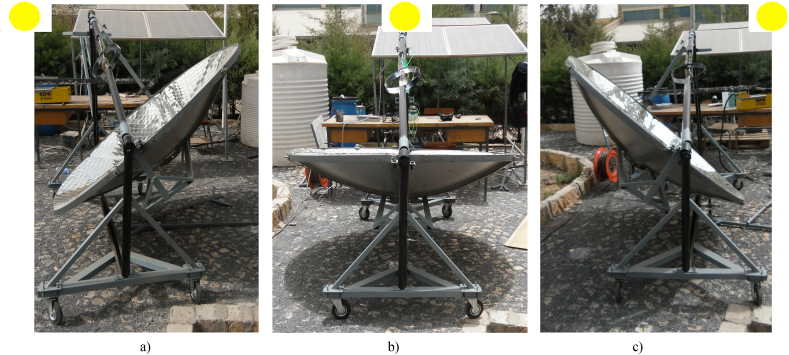
Daily solar radiation tracking (a) sunset (b) solar noon (c) sunrise time.

The total rotation of the vertical reference line Y–Y is 75°, which means that it rotates a maximum of 37.5° for the morning (Fig. 11a) and also for the afternoon (Fig. 11c), to track the sun for 5 h per day During the solar noon at the time of intersection, X-X and Y–Y line (Xy) parabolic dish central axis will be parallel with solar noon axis and the altitude angle will be high (see Fig. 11b) During the tracking process, the parabolic dish was secured to the hanging support with a bolt and nut to prevent the structure from slipping as shown in Fig. 10.

**Fig. 12:**
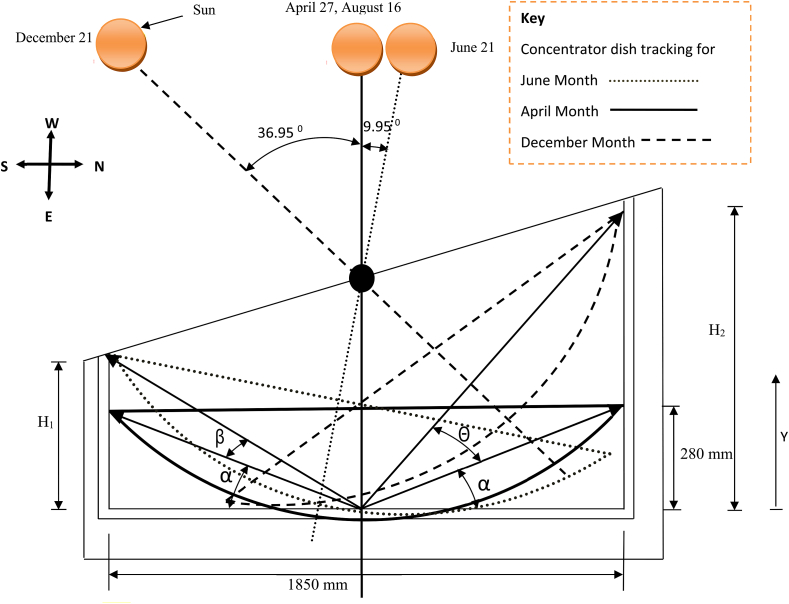
Monthly Solar radiation tracking (concentrator parabolic dish side view).

#### 4.1.3 Monthly manual tracking

Using a latitude of 13.5° N and sun declination angles of 23.45° and −23.45°, the minimum and maximum solar noon altitude angles for Mekelle city are 53.05° and 99.95°, respectively using Eqs. (3) and (4) and clearly shown on Table 2. This means that on December 21 and June 21, the central axis of the solar parabolic dish concentrator will make a maximum of 36.95° to the south and 9.95° to the north from the vertical axis, as shown in Fig. 12.

Equation (4) was used to calculate the solar noon altitude angle for Mekelle city on June 21 and December 21. The first task completed for monthly solar radiation was identifying the vertical column high of the hanging structure, and in order to do that the first angle calculated was α, using Eq. (6),

(6) Tan(α)=280*2/1850=0.303

$$\alpha =16.84^{0}$$

The value of angle β and θ is solar noon altitude angle at June 21 and December 21 for 13.5° N latitude i.e., 9.95° and 36.95° respectively. The following formulas (Eqs. (7) and (8)) can be used to calculate the vertical length of both columns.

(7) $$H_{1}=\tan\left(16.84+9.95\right)*925\;mm$$

$$H_{1}=467mm$$

And, for the second vertical height,

(8) $$H_{2}=\tan\left(16.84+36.95\right)*925\;mm$$

$$H_{2}=1263\;mm$$

**Fig. 13:**
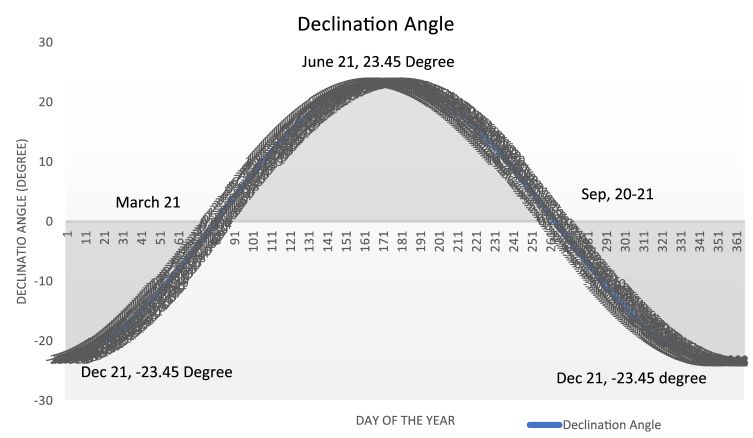
Declination of the sun for each day of the year.

To find the month or, more specifically, the day when the solar altitude angle is 90° at solar noon (the time when the sun is over the center of the concentrator dish), we must first calculate the declination angle at those months (days). So, for α_n_ = 90°, the declination angle is calculated using Eq. (4). And this declination angle is found two times in a year as we can see from Fig. 13 and Table 2. This Time is more specifically between spring equinox and fall equinox on April 27 and August 16 as we can see from Table 2, Figs. 12 and 13.

$$\alpha_{n}=900-L+\delta$$

90°=90°−13.5°+δ

δ = 13.50

The day number at this declination angle was determined using the declination angle formula described in Eq. (3). As a result, at N = 117 or April 27, the value of δ = 13.62°, close to 13.5° of declination angle is obtained. And the declination angle at N = 228 is = 13.45° on August 16. Fig. 13 depicts the declination angle for each day of the year. N = 116.5 (April 27) and N = 228 (August 16) are the two days when the sun will be overhead on the center of the concentrator at solar noon for this specific location.

So, to find the vertical distance arrangements of the concentrator dish in the hanging metal structure with solar radiation at solar noon for different months of the year, the only thing that will change is the solar declination angle (δ). Using a solar declination angle for 2 weeks of a month the vertical position of the concentrator dish relative to lower horizontal bar is calculated as follow in Table 2, using Eqs. (3), (4), (7) and (8).

According to Table 2 the parabolic dish moves vertical axis to the north direction for the month of May, June, July and August (negative with the vertical axis) and for the rest of the month the parabolic concentrator dish face to the south hemisphere.

#### 4.1.4 Arrangement of the field test setup

**Fig. 14:**
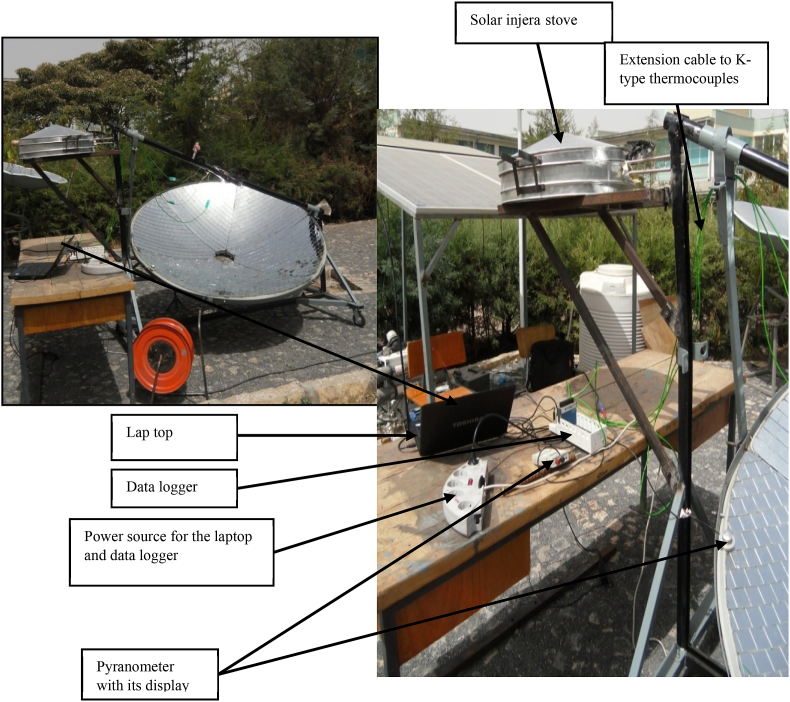
Field test setup for data acquisition.

The field test setup was seated using the manual tracking method described above for daily and monthly solar angle variation. All of the setup was required before testing the system for temperature at any interested point of the system, pressurized steam generation in closed circulation, including under the solar baking Injera with the system. This test made use of the following laboratory equipment (see Fig. 14):

• The whole system, i.e., concentrator parabolic dish, receiver, Stainless Steel piping system, external structure support, solar injera baking stove and its' support.
• Lenovo CORE i7 vPro Lap top for data acquisition, with Lab VIEW Express signal 2009 software and National Instruments data logger
• K-type thermocouples with extension cables.
• METEON Pyranometer with its display

#### 4.1.5 Solar radiation and temperature correlation at different point of the receiver

**Fig. 15:**
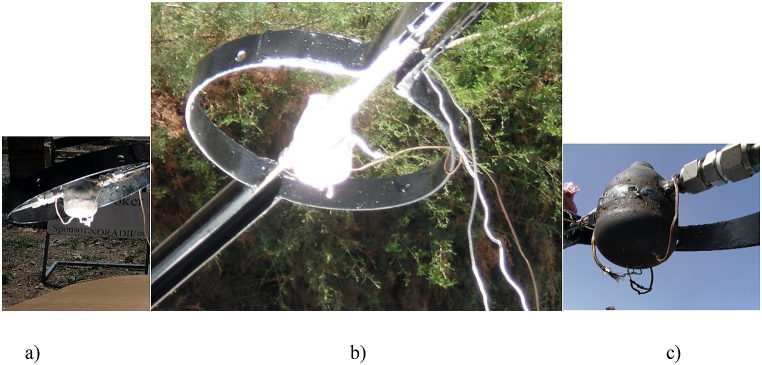
Data collection point on the receiver (a) one point on the center (b) and (c) three points on the receiver.

**Fig. 16:**
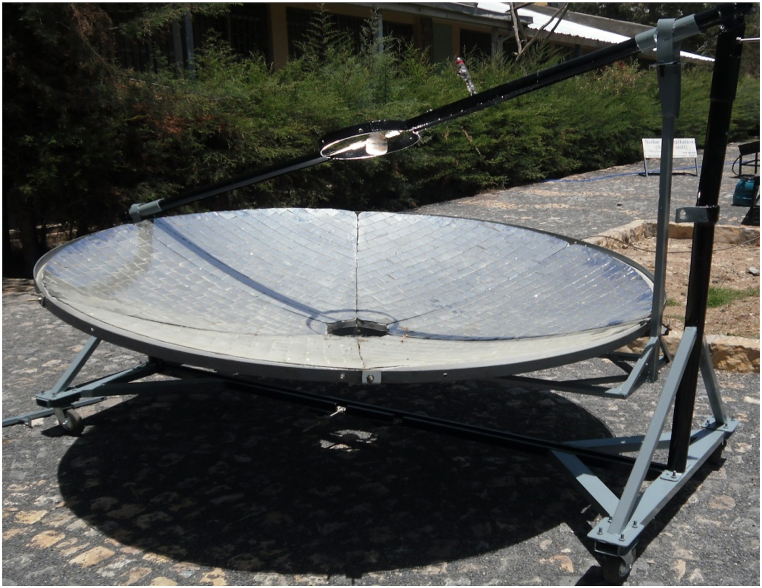
Tracking system, the hanging support structure track the sun using concentrator, solar noon time.

An experimental dataset was collected to determine whether manual tracking is working properly as expected. To collect data for this purpose, a solar irradiation using a pyranometer and temperature data using a K- type thermocouple with extension cable and National Instrument Data logger integrated with Lab VIEWS Signal Express were used. In the first experiment, one receiver point is used to compare the two data sets. In the second experiment, three points were used to compare the variation in solar irradiation with the variation in temperature on the receiver's surface (see Fig. 15). These correlations can demonstrate a clear relationship of different receiver temperature data with the orientation of the manual tracking mechanism, tracking ability to track the sun path along the receiver's surface. On the first experiment one point on the lower part of the receiver was selected, but on the next experiment three point was selected for temperature data on the lower part, to the east, middle and west direction (see Fig. 15(a,b,c) and Fig. 16).

When the solar ray concentrated on the receiver it looks like Fig. 15(a and b). The solar radiation that reflects from the parabolic concentrator mirror dish concentrate on the receiver and it heats up the water inside the receiver to generate steam. To read the daily solar radiation on the concentrator, the pyranometer was positioned on the concentrator with the axis parallel to the axis of solar ray and receiver center of focus (see Fig. 14). This position helps to read the exact solar radiation that comes to the concentrator in (W/m^2^), according to American Society of Heating, Refrigerating and Air-Conditioning Engineers (ASHERA) 92–1986 test set up.

**Fig. 17:**
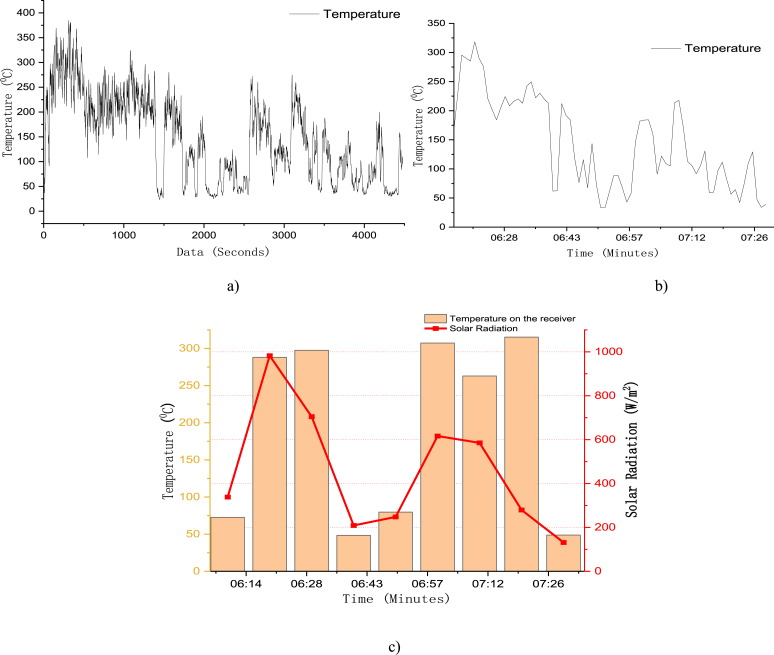
(a) A second temperature data on a receiver (b) 1 min average temperature reading on a receiver (c) 10-min solar radiation of May 22/2012 and that time temperature on the receiver.

The manual tracking system track the sun by moving from west to east to track the sun while moving from east to west as shown from Fig. 11. The solar radiation on May 22/2012 was not good, there was an intermittent cloud cover. As a result of this, the temperature result on the receiver was not good. The solar radiation distribution on the day of May 22/2012 from 12:10 p.m. to 1:30 p.m. is shown on Figure Fig. 17(c). The average solar radiation of this recorded data was 647 W/m^2^.

From Fig. 17, the solar radiation was taken in 10 min time interval and the variation of solar radiation was fluctuating quickly, this makes the temperature on the receiver vary a lot. The maximum temperature obtained at this day was 384.82 °C on the surface of the receiver with 1 s data interval obtained using the Lab View Signal Express 2009 data collecting software (see Fig. 17(a)) but when this 1 s data was converted to 1 min average and 10 min the maximum temperature value was 318 °C and 290 °C respectively (see Fig. 17(b and c)). The average temperature at the receiver for 10-min data was 134 °C. The correlation coefficient of the 10-min solar radiation and the temperature on the receiver for this experiment was r = 0.73 that indicate there is strong positive relation between the solar radiation and the temperature on the receiver.

**Fig. 18:**
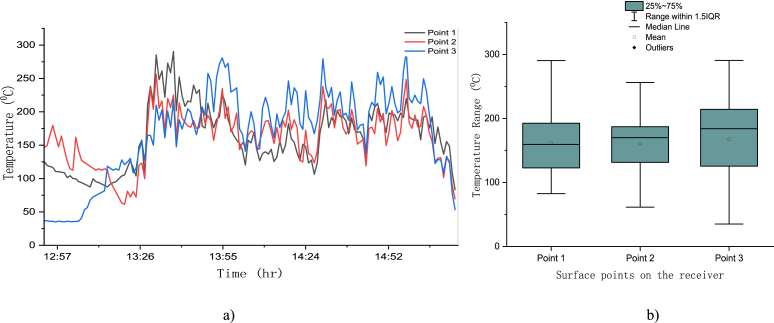
(a) Three-point receiver surface temperature (b) box plot for three-point receiver surface temperature.

**Fig. 19:**
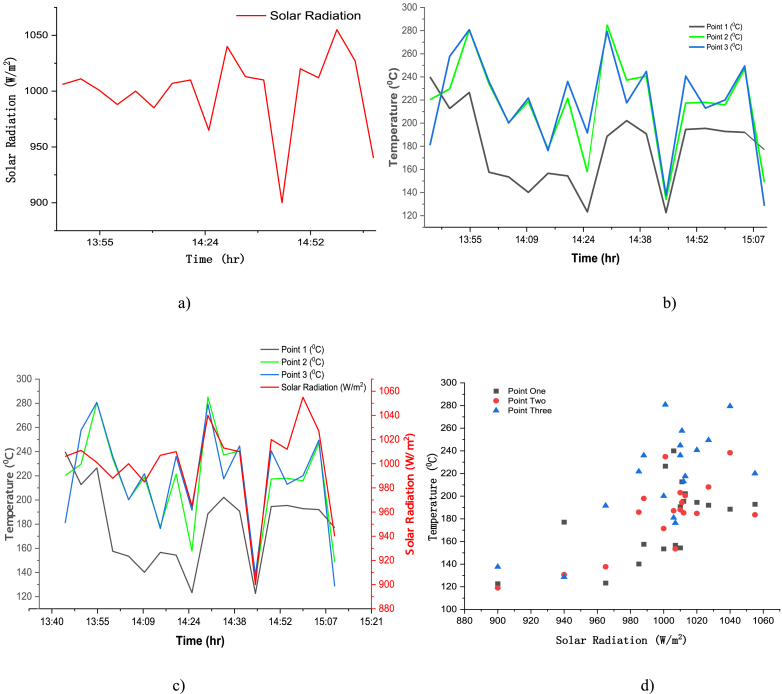
(a) Solar radiation profile (b) temperature distribution of three point on receiver surface (c) patter match of solar radiation and temperature of the receiver surface (d) scatter plot shown linear relation of solar radiation and temperature on the receiver.

The second without load test was taken on May 29, 2012. On the second test, the temperature distribution on three points of the receiver was recorded and the temperature distribution was uniform on those points. The data was recorded from 6:45 p.m. tile 3:15 p.m. for about one and half hour (see Fig. 18(a). The point at which the solar radiation was concentrated experience a maximum temperature of 350 °C with 1 s time interval but for the case of 1 min average, the maximum temperature was 290 °C for point one and three (see Fig. 18(a and b)) and for five -minute temperature scale, it was around 281 °C for the third point from 1:45 p.m. to 3:10 p.m. During this data acquisition, solar radiation was also recorded, as shown on Fig. 19(a). The average temperature of these three points was in the range of 161 °C to 167 °C as shown on Fig. 18(b).

Form Fig. 18(a) the temperature profile of point 1, 2 and 3 indicate the right position of the tracking system. Point 1 was position on the lower west side of the receiver and this point was having the highest temperature before solar noon and in the afternoon this temperature declines as compared to point 2 and 3. Since point two is positioned on the lower most point of the receiver, the temperature profile of point 2 is in between those two points. Similarly, because point 3 is positioned on the east lower location of the receiver the temperature profile is lower before solar noon and its value increased and placed above all those two points after solar noon as it can be clearly seen from Fig. 18(a).

The solar radiation was in the range of 900–1040 W/m^2^, and the average solar radiation was 999 W/m^2^ as shown in Fig. 19(a). The temperature profile of 5 min for point one was below point two and three in the afternoon since this point was located on the west side of the receiver lower point, in which the temperature profile of this point was higher than that of point two and three before the solar noon (see Fig. 19(b and c)). Hence, from the scatter plot the highest temperature for the highest solar radiation was obtained on those three points of the receiver surface (see Fig. 19(d)).

To see the relationship of the solar radiation with the temperature profile of those different points on the receiver, and to test if whether the manual tracking mechanism is properly tracking the sun path for each 5 min of the data collection, a correlation was calculated to find out the coefficient of correlation using Eq. (9). The Pearson product moment correlation assesses the degree of linear relationship between two quantitative variables and correlation measures the strength of the relationship between two variables, temperature on the surface of the receiver and the solar radiation from the sun [21].

(9) $$r=\frac{\sum \left(T_{i}-T_{mean}\right)\left(I_{i}-I_{mean}\right)}{\sqrt{\sum {\left(T_{i}-T_{mean}\right)}^{2}\sum {\left(I_{i}-I_{mean}\right)}^{2}}}$$

**Fig. 20:**
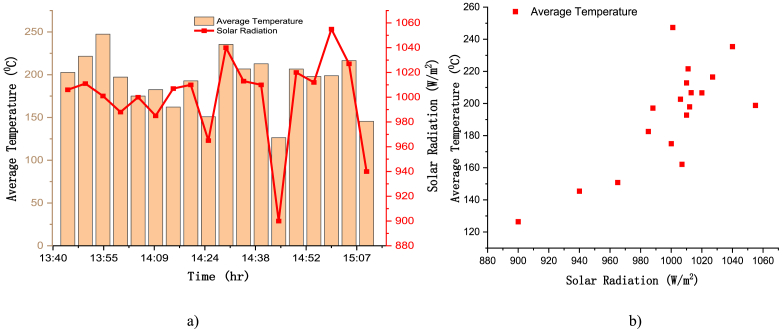
Average temperature versus solar radiation (a) two variables per time (b) scatter plot of temp versus solar radiation.

The average temperature of three points on the receiver has a correlation coefficient value of r = 0.766 with the solar radiation (see Table 3), which is stronger positive correlation. The average temperature of those three points with the solar radiation are shown on Fig. 20(a). These two variables have a positive correlation coefficient with linear relationship as it is explicitly shown on Fig. 20(b). And, the reason why the correlation for point one is lower is because of its position relative to the position of the sun, the sun position during the experiment was around solar noon and move to the west during 1 h and 25 min which gave high temperature to point 3 located on the east side of the receiver, but point one was on the east part of the receiver lower part that received solar radiation during the morning time (see Table 3).

**Table 3:** Correlation Coefficients between Solar radiation and different point temperature on the receiver surface.

		Correlation of Each point temperature with Solar irradiation	
Correlation of test one			0.726
Correlation of point 1 and 2	0.625	Point 1 and Solar radiation	0.564
Correlation of point 1 and 3	0.426	Point 2 and Solar radiation	0.740
Correlation of point 2 and 3	0.914	Point 3 and Solar radiation	0.708
Correlation of average temp of three points with Solar Radiation			0.766

## 5 Conclusion

Solar base energy producing equipments must have a tracking mechanism, so that it will track the sun along its path. The energy production depends on concentration ratio of the solar apparatus as the concentration ratio increased the apparatus require a double axis tracking. Parabolic concentrator is a double axis solar collector which track the sun daily and seasonally. Hence, this kind of solar apparatus require a two-axis tracking which highly relay on the tracking mechanism. A point receiver requires a precise tracking mechanism so that the sun will concentrate on it.

On this paper, a double axis manual tracking mechanism is designed and manufactured. The tracking system track the sun seasonally for the whole year and it can track the sun for 75° of hour angle daily. The daily tracking system can track the sun for a maximum of 37.50° in the morning and in the afternoon, to track the sun for 5 h per day, two and half in the morning and two and half in the afternoon. During the tracking process, the parabolic dish was secured to the hanging support with a bolt and nut to prevent the structure from slipping. The system will track the path of the sun every 10 min.

The manual tracking system was tested to examine whether the tracking system track the sun path along a fixed receiver point. According to this, the correlation of the solar radiation at a particular time was evaluated as compared to the temperature of the receiver surface. On the first experiment a point temperature on the lower point of the receiver surface was measured and it was compared with the solar radiation, a correlation coefficient r = 0.726 was obtained. On the second test for an average temperature of three points on the receiver, a correlation coefficient of r = 0.766 has found with solar radiation. Both of the experiment shows there is a strong positive correlation between the solar radiation and temperature on the surface of the receiver which indicate the manual tracking track the sun properly, even if the temperature measurement was collected discretely from different point of the receiver's surface as compared to the continues data of the solar radiation in which one receiver surface point with low temperature at some time of the day was compromised with high temperature of other point on the opposite position.

## Author contribution statement

Arkbom Hailu Asfaw: Conceived and designed the experiments; Performed the experiments; Analyzed and interpreted the data; Contributed reagents, materials, analysis tools or data; Wrote the paper.

## Funding statement

This work was supported by Ethiopian Institute of Technology – Mekelle EiT – M as part of the (NORAD's Master Program in the Energy and Petroleum Sector) at the Department of Mechanical Engineering (EnPe Project).

## Declaration of interest's statement

The author declares no competing interests.

## Data availability statement

Data included in article/supplementary material referenced in article.

## List of symbols

α: Solar Altitude angle
φ: Solar Zenith angle
L: Latitude
δ: Solar declination angle
z: Solar Azimuth angle
h: Hour angle
r: correlation coefficient
T_i_: individual values of the Temperature
T_mean_: mean of the values of the Temperature
I_i_: individual values of the Solar irradiation
I_mean_: mean of the values of the Solar irradiation
